# Vasopressin impairs brain, heart and kidney perfusion: an experimental study in pigs after transient myocardial ischemia

**DOI:** 10.1186/cc6794

**Published:** 2008-02-21

**Authors:** Stig Müller, Ole-Jakob How, Stig Eggen Hermansen, Thor Allan Stenberg, Georg Sager, Truls Myrmel

**Affiliations:** 1 Laboratory of Surgical Research, Institute of Clinical Medicine, University of Tromsø, N-9037 Tromsø, Norway; 2 Department of Cardiothoracic and Vascular Surgery, University Hospital North Norway, Norway; 3 Department of Pharmacology, Institute of Medical Biology, University of Tromsø N-9037 Tromsø, Norway

## Abstract

**Introduction:**

Arginine vasopressin (AVP) is increasingly used to restore mean arterial pressure (MAP) in low-pressure shock states unresponsive to conventional inotropes. This is potentially deleterious since AVP is also known to reduce cardiac output by increasing vascular resistance. The effects of AVP on blood flow to vital organs and cardiac performance in a circulation altered by cardiac ischemia are still not sufficiently clarified. We hypothesised that restoring MAP by low dose, therapeutic level AVP would reduce vital organ blood flow in a setting of experimental acute left ventricular dysfunction.

**Methods:**

Cardiac output (CO) and arterial blood flow to the brain, heart, kidney and liver were measured in nine pigs using transit-time flow probes. Left ventricular pressure-volume catheter and central arterial and venous catheters were used for haemodynamic recordings and blood sampling. Transient left ventricular ischemia was induced by intermittent left coronary occlusions resulting in a 17% reduction in cardiac output and a drop in MAP from 87 ± 3 to 67 ± 4 mmHg (p < 0.001). A low-dose therapeutic level of AVP (0.005 U/kg/min) was used to restore MAP to pre-ischemic values (93 ± 4 mmHg).

**Results:**

AVP further impaired systemic perfusion (CO and brain, heart and kidney blood flow reduced by 29, 18, 23 and 34%, respectively) due to a 2.0-, 2.2-, 1.9- and 2.1-fold increase in systemic, brain, heart and kidney specific vascular resistances. The hypoperfusion induced by AVP was associated with an increased systemic oxygen extraction. Oxygen saturation in blood drawn from the great cardiac vein fell from 29 ± 1 to 21 ± 3% (p = 0.01). Finally, these effects were reversed 40 min after AVP was withdrawn.

**Conclusion:**

Low dose AVP induced a pronounced reduction in vital organ blood flow in pigs after transient cardiac ischemia. This indicates a potentially deleterious effect of AVP in patients with heart failure or cardiogenic shock due to impaired coronary perfusion.

## Introduction

Synthetic arginine vasopressin (AVP) has been increasingly used to restore systemic blood pressure in low pressure shock states unresponsive to conventional inotropes [1,2]. The association found between low plasma AVP levels and advanced vasodilatory shock has given a rationale for administrating this hormone in septic shock and in systemic inflammatory response syndrome (SIRS) [1-4]. AVP has also been given to patients in post-cardiotomy cardiac failure [1] and in cardiogenic shock complicating myocardial infarction [5]. Interestingly, the modulating effect of AVP on cardiac index (CI) seems to lower CI in hyperdynamic patients whereas an elevation of CI has been found in hypodynamic patients [6]. AVP treatment in patients with low or normal CI is however controversial, since experimental studies [7-9] and small clinical trials [10-13] have shown that the hormone may compromise cardiac output and organ perfusion due to its vasoconstrictive effect.

The response in vital organ perfusion, that is, pulmonary, coronary and cerebral circulation, to AVP-infusions is still insufficiently clarified [14]. From its basic physiological effect on V1-receptor stimulation in vascular smooth muscle [14], one would predict an overall vasoconstrictive effect after AVP infusion. However, infusion of the drug has been found to dilate the coronary, pulmonary and vertebrobasilary arteries [14,15].

The aim of the present study was to asses whether infusion of AVP in a dose at therapeutic level in pigs after transient cardiac ischemia would reduce coronary, cerebral, renal and total organ blood flow (cardiac output). As the treatment with AVP after cardiac ischemia is potentially the most deleterious of conditions if vasoconstrictive in vital organs, we elected to assess the vascular response after reperfusion of an ischemic condition in the pig heart.

## Materials and methods

### Animals

The experimental protocol was approved by the local steering committee of the National Animal Research Authority (NARA). Nine castrated male domestic pigs (cross breed of Norwegian Landrace and Yorkshire pigs) weighing 30 ± 1 kg were adapted to the animal department for 5–7 days and fasted overnight before the experiment with free access to water.

### Instrumentation

The animals were pre-medicated with intramuscular injections of 20 mg/kg ketalar (Pfizer AS, Norway) and 1 mg atropine (Nycomed Pharma, Norway). Anaesthesia was induced by intravenous injection of 10 mg/kg pentobarbital-sodium (Abbott, Sweden) and 0.01 mg/kg fentanyl (Hameln Pharmaceuticals, Germany), and the animals were normoventilated after tracheotomy. A central venous catheter was placed through the left internal jugular vein, and anaesthesia was maintained throughout the experiment using a continuous infusion of 4.0 mg/kg/h pentobarbital-sodium, 0.02 mg/kg/h fentanyl and 0.3 mg/kg/h midazolam (B. Braun, Melsungen, Germany). The circulating volume was maintained by a 20 ml/kg/h continuous infusion of 0.9% NaCl supplemented with 1.25 g/l glucose. The animals received 2 500 IU heparin, and 5 mg/kg amiodarone (Sanofi-Synthelabo, Sweden) to avoid blood clotting of catheters and cardiac arrhythmias. Central venous pressure (CVP) was monitored through the right jugular vein. Measurement of mean arterial pressure (MAP) and arterial blood sampling in the abdominal aorta was performed through a catheter inserted through the left femoral artery. After sternotomy, the left hemiazygos vein was ligated to avoid return of systemic blood to the coronary sinus. Transit time flow probes (CardioMed CM-4000, Medi-Stim AS, Horten, Norway) were placed on the left anterior descending coronary, the right carotid artery and the pulmonary artery in order to measure changes in coronary blood flow, cerebral blood flow and cardiac output, respectively. A 7 Fr balloon catheter was introduced to the inferior caval vein for preload reduction. Also, a 7 Fr dual field, combined pressure-conductance catheter (CD Leycom, Zoetermeer, the Netherlands) was inserted into the left ventricular cavity via the left carotid artery for measurements of left ventricular pressure and volume (P-V). Assessment of the individual segments of the pressure-volume loops confirmed the proper placement of the catheter. Myocardial venous blood was drawn from a catheter placed in the great cardiac vein via the coronary sinus. A catheter was inserted into the main pulmonary trunk through the right ventricular wall for measurement of mean pulmonary artery pressure (MPAP) and central venous oxygen saturation (sVO_2_). Thereafter, a midline laparotomy was performed and the urine bladder was catheterised via a cystotomy. Transit time flow probes were placed on the main hepatic artery and left renal artery in order to measure liver and kidney blood flow, respectively. The animals were allowed to rest for 1 h before baseline measurements.

### Experimental protocol

Full data sets were collected five times during a complete experiment. Baseline values were recorded 1 h after the end of instrumentation. The ischemia/reperfusion protocol was then implemented during the next 30–50 min (see below). Post-ischemic values were recorded 40 min after the end of the ischemia/reperfusion protocol. The pigs were subsequently given 0.005 U/kg/min AVP for 40 min as an initial dose (in a series of pilot experiments this was found to be the lowest dose necessary to restore blood pressure to pre-ischemic values). The third recordings were then performed (AVP-initial). Thereafter, the AVP infusion rate was individually adjusted to keep the mean arterial pressure stable at approximately 90 mmHg. The fourth measurements (AVP-90 mmHg) were carried out 40 min into the infusions of this individual AVP dose. Finally, the AVP was withdrawn and the last measurements (Withdrawal) were performed 40 min thereafter. Obtaining each data set required approximately 3 min and the samples were carried out in the following order: 1, blood sampling from femoral artery, pulmonary artery and great cardiac vein; 2, blood oxygen levels from femoral artery (arterial or artO_2 _sat), pulmonary artery (central venous or SVO_2_) and great cardiac vein (sinus coronarius or sin cor O_2 _sat); 3 the respirator was then disconnected for 10 s to avoid pressure influence on haemodynamics, and steady state P-V data, MAP, MPAP, CVP, carotid artery flow, renal artery flow, LAD flow, hepatic and pulmonary artery flows were recorded. After a further period of 1 min, the respirator was disconnected for another 10 s and the balloon catheter in the inferior caval vein was inflated to obtain P-V data during vena cava occlusions (VCO) in accordance with previous protocols from our laboratory [16].

### Ischemia reperfusion protocol

The ischemia reperfusion protocol has in principle been described previously [17]. The present modified protocol was based on repeated coronary occlusions to induce an acute impairment of cardiac performance, and aimed to reversibly reduce the flow in the larger part of the left ventricle but to avoid the arrhythmias and pump failure connected to prolonged coronary occlusions. To create coronary flow perturbations, rubber bands were looped around the LAD and circumflex coronary arteries. The bands were tightened sufficiently to demonstrate zero flow in the LAD signal during occlusions. The protocol was initiated by two occlusion periods of 60 s followed by 120 s of reperfusion. The following occlusions were guided by a maximum occlusion time of 120 s or were terminated if MAP fell below 30 mmHg during occlusions. The reperfusion time between occlusions were 30–120 s with the next occlusion starting at the onset of maximal hyperaemia. The cumulative ischemic time was 20 ± 4 min, and was terminated when MAP recovered to only 90% of baseline. This protocol resulted in a slightly more pronounced post-ischemic hypotension compared to our previous study [17]. In four pigs, defibrillation was necessary to resuscitate ventricular arrhythmias occurring during the LAD/CX occlusions.

### Biochemical analyses

Blood gases and base excess were immediately analysed on a blood gas analyser (Rapid lab, Chiron Diagnostics, Emeryville, CA, USA). Blood samples for the other analyses were put on ice, quickly centrifuged and plasma was then frozen. Plasma lactate levels were determined on a biosensor (ABL 800, Radiometer, Bergman Diagnostica, Lillestrom, Norway), while troponin T was measured by electrochemiluminiscense (Modular E, Roche, Diagnostics, Basel, Switzerland). Plasma AVP was analysed using an immunoassay kit (Assay Designs, Ann Arbor, MI, USA). Sampling, preparation and analysis of plasma catecholamines were carried out as previously described [18,19] with minor modifications. Noradrenaline and adrenaline were separated by HPLC (Dionex P680, Dionex ASI-100, Chromsystems analytical column and eluent) and their concentrations determined with an electrochemical detector (ESA Coulochem III).

### Calculation of haemodynamic indices

Vascular resistance in various systemic beds were calculated as the pressure drop (MAP-CVP) divided by the arterial blood flow to the respective organs. Data from the pressure-volume catheter was analysed on CircLab (GTX Medical Software, Zoetermeer, the Netherlands) to obtain various indices of ventricular function. Calibration and practical use of the left ventricular P-V catheter has been described in detail previously [16]. Absolute volume assessment by the conductance technology requires an estimate of parallel conductance. This is performed by hypertonic saline infusion. In the present study, injection of hypertonic NaCl was not employed since a hypertonic coronary flush could influence the coronary response to AVP in the experimental protocol. Calculations of absolute ventricular volumes were therefore not performed. Such calculations would have demanded an alternate technique such as echocardiography.

The first derivative of ventricular pressure (dP/dt) was recorded as maximum and minimum values. The time-constant of isovolumic relaxation (Tau) was calculated according to Mirsky [20]. The slope of EDPVR and ESPVR denotes the line drawn through the end diastolic and end systolic pressure volume relationship for a family of PV-loops obtained during an abrupt vena cava occlusion [21]. The preload recruitable stroke work index (PRSWi) integrates the systolic and diastolic performances as the relation between stroke work (SW) and end diastolic volume (EDV).

### Statistics

The data are expressed as mean ± SEM. A one-way repeated measures analysis of variance (ANOVA), followed by the Holm-Sidak test, were used to determine differences in the series of interventions. Data obtained 40 min after the ischemia/reperfusion protocol were regarded as reference value. We labelled significant differences between all data points as compared to this reference value. In addition, we labelled significant differences between (AVP-90 mmHg) and (Withdrawal). Differences between means were regarded as statistically significant when p values were less than 0.05.

## Results

### Post-ischemic circulation

**Figure 1:**
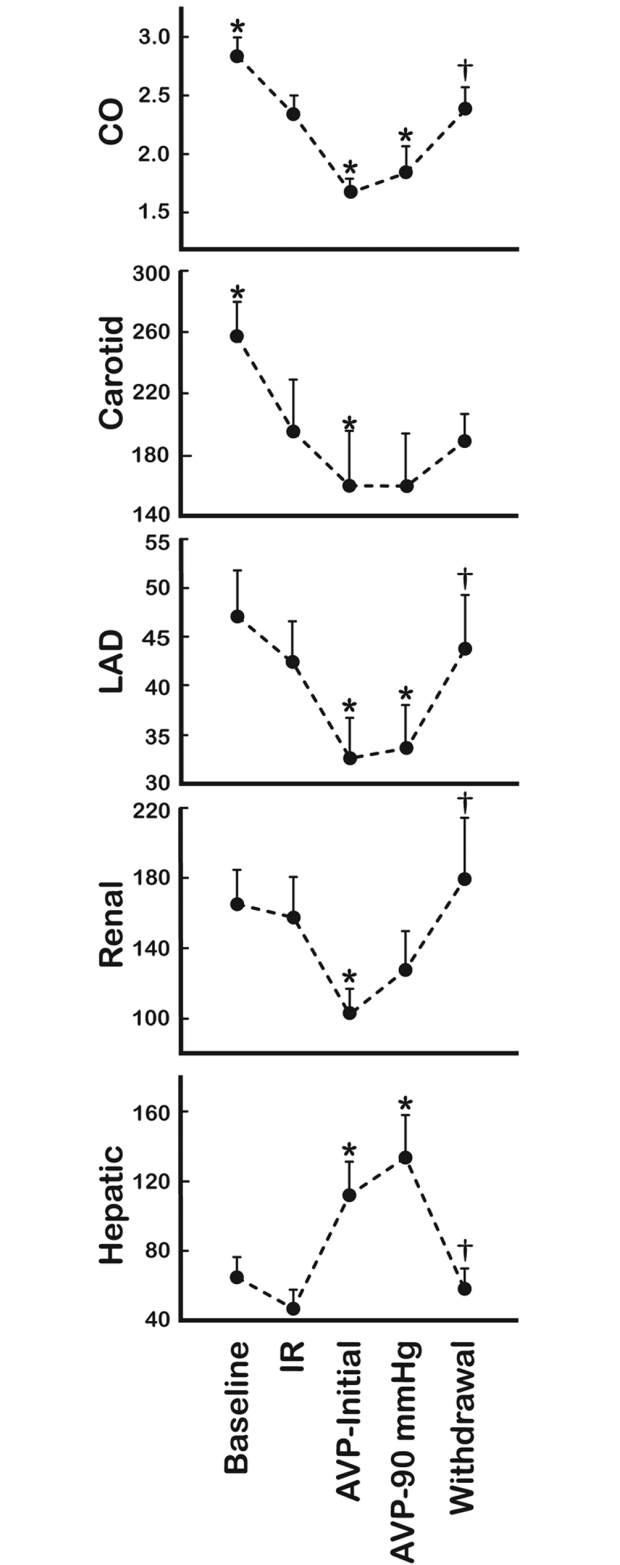
Flow recordings. Data in mL/min (L/min for CO) obtained at baseline, after ischemia/reperfusion (IR) protocol, initial AVP infusions (AVP-initial), mean pressure of 90 mmHg (AVP-90 mmHg) and after drug withdrawal (Withdrawal) (*n *= 9). CO, cardiac output; carotid, right carotid artery; LAD, left anterior descending; Renal, left renal artery; Hepatic, main liver artery. *Significant difference from (AHF); †significant difference (AVP-90 mmHg) and (Withdrawal).

**Figure 2:**
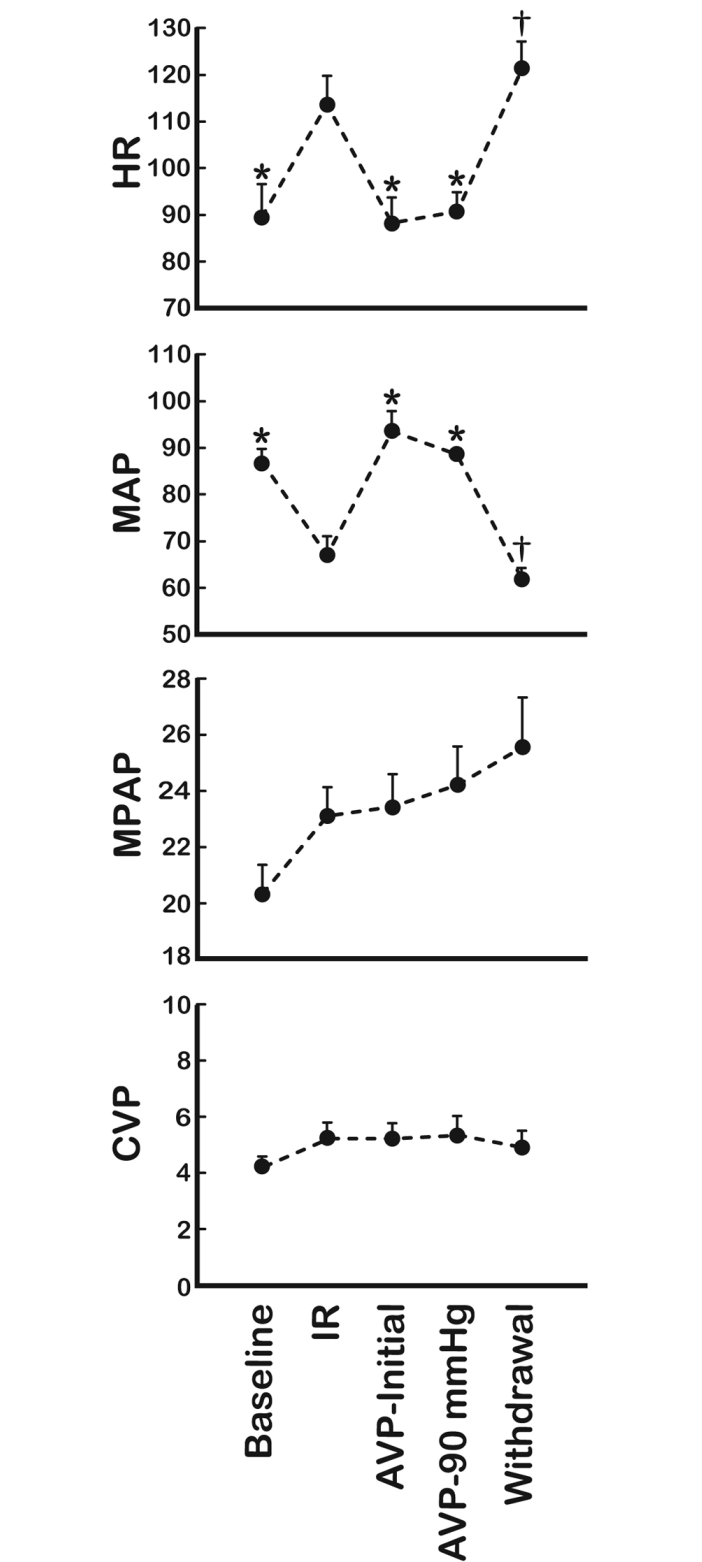
Heart rate (HR) and pressure recordings. Data obtained in mmHg at baseline, after ischemia/reperfusion (IR) protocol, initial AVP infusions (AVP-initial), mean pressure of 90 mmHg (AVP-90 mmHg) and after drug withdrawal (Withdrawal) (see text for details) (*n *= 9). MAP, mean arterial pressure; MPAP, mean pulmonary arterial pressure; CVP, central venous pressure. *Significant difference from (IR); †significant difference between (AVP-90 mmHg) and (Withdrawal).

**Figure 3:**
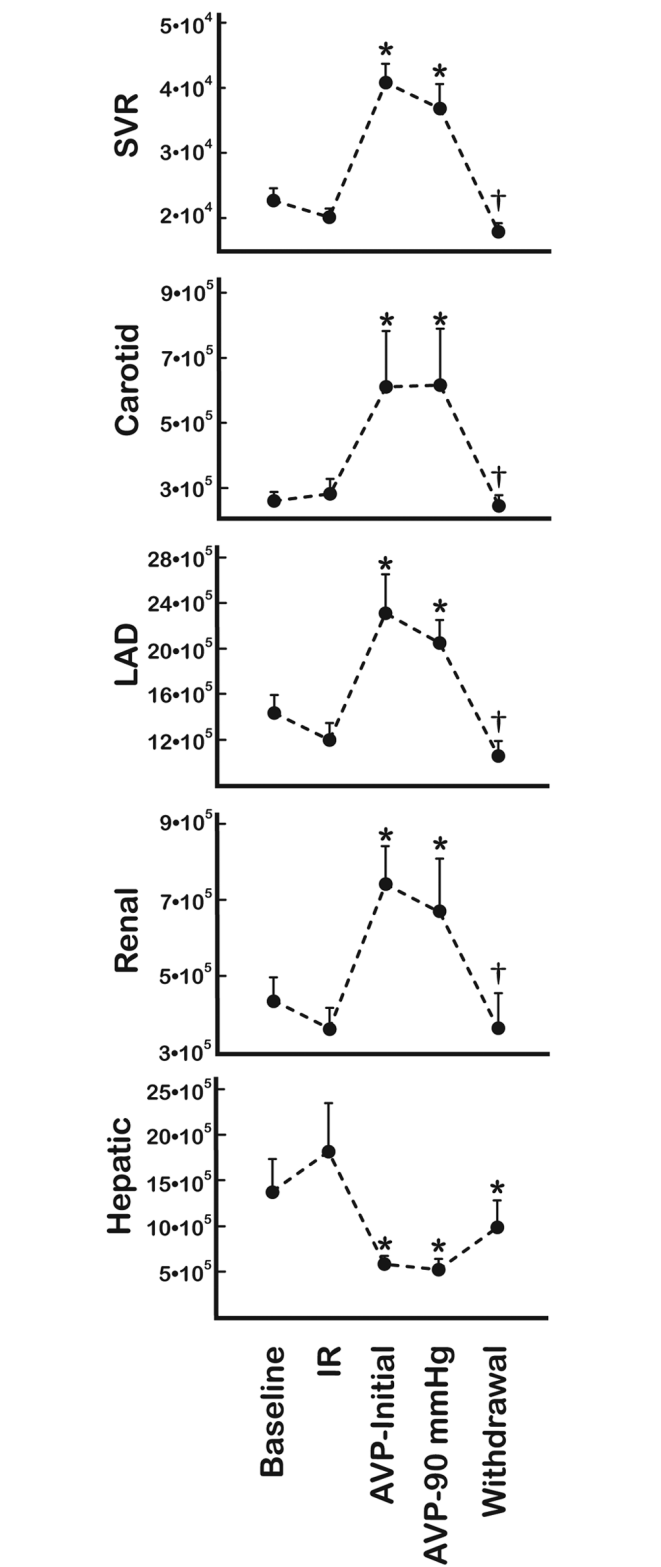
Vascular resistance. Data in dynes/sec/cm^5 ^are obtained at baseline, after ischemia/reperfusion (IR) protocol, initial AVP infusions (AVP-initial), mean pressure of 90 mmHg (AVP-90 mmHg) and after drug withdrawal (Withdrawal) (*n *= 9). The values are calculated as (MAP-CVP)/(specific organ flow). SVR, systemic vascular resistance; Carotid, specific resistance in right carotid artery; LAD, specific resistance in left anterior descending artery supplying left ventricle; Renal, specific resistance in main artery supplying left kidney; Hepatic, specific resistance in main liver artery. *Significant difference from (IR); †significant difference between (AVP-90 mmHg) and (Withdrawal).

**Figure 4:**
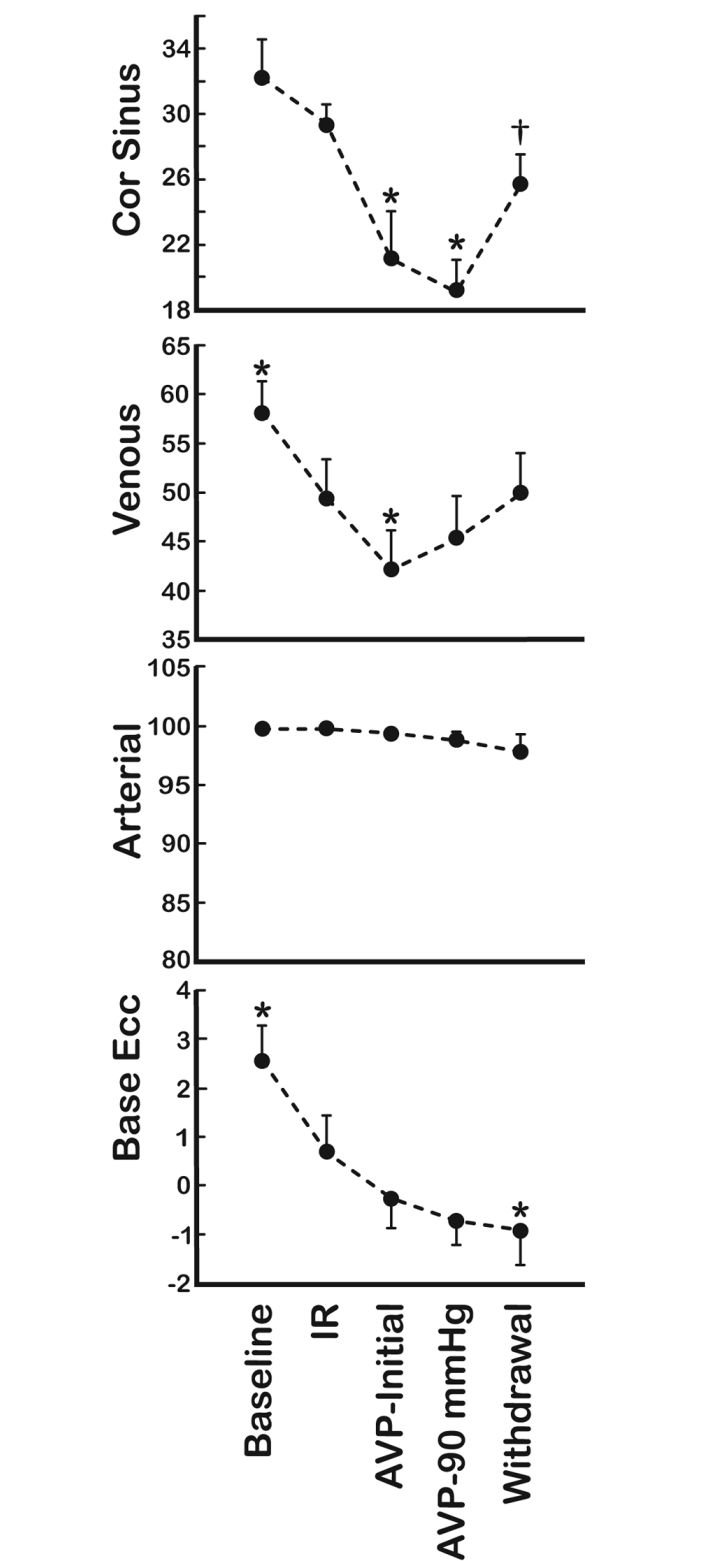
Blood gas analysis. Data obtained at baseline, after ischemia/reperfusion (IR) protocol, initial AVP infusions (AVP-initial), mean pressure of 90 mmHg (AVP-90 mmHg) and after drug withdrawal (Withdrawal) (*n *= 9). Cor sinus, Venous and Arterial are (%) oxygen saturation in blood obtained from the main heart vein, pulmonary artery and femoral artery, respectively. Base excess was calculated from systemic arterial blood. *Significant difference from (IR); †significant difference between (AVP-90 mmHg) and (Withdrawal).

**Figure 5:**
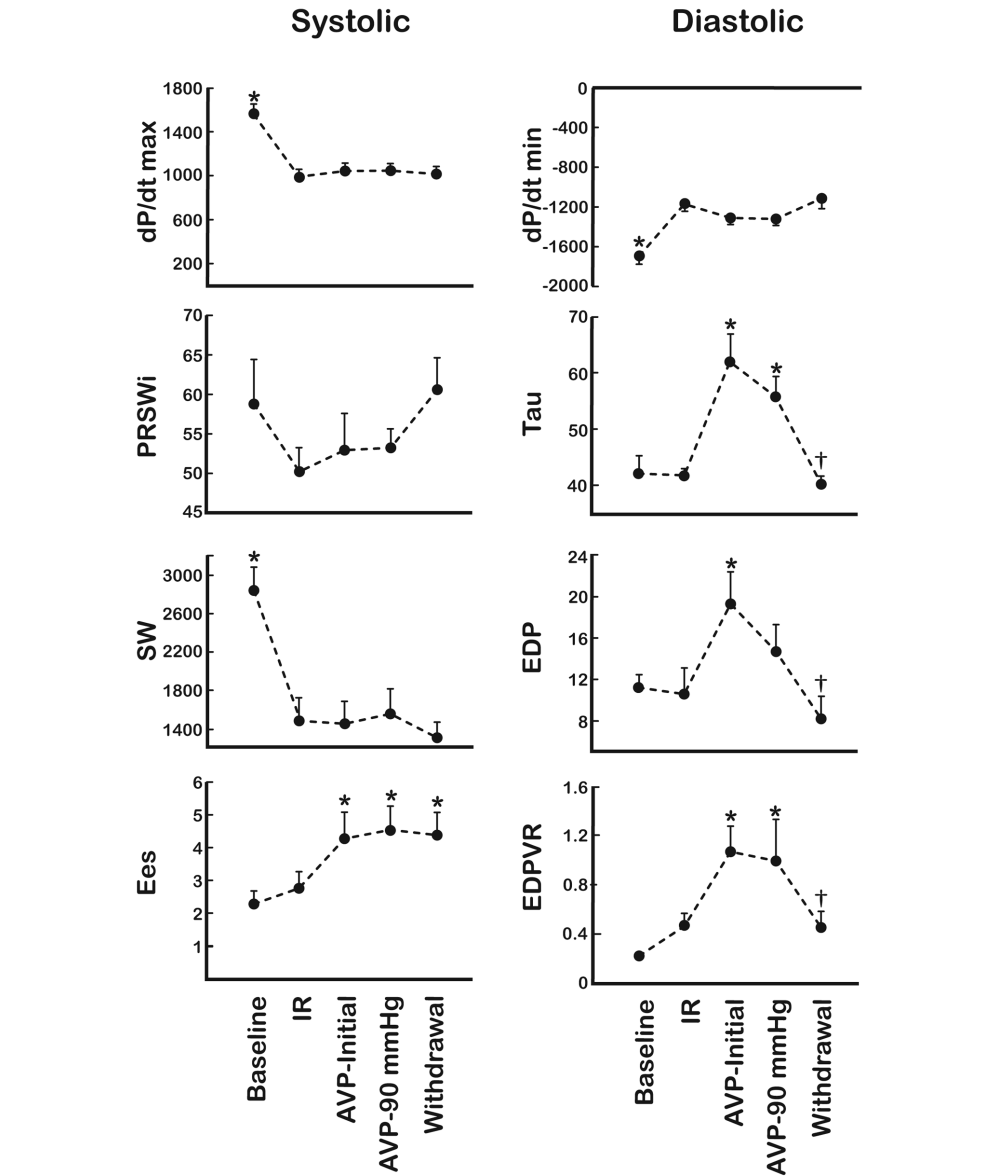
Indices of left ventricular function. Data obtained at baseline, after ischemia/reperfusion (IR) protocol, initial AVP infusions (AVP-initial), mean pressure of 90 mmHg (AVP-90 mmHg) and after drug withdrawal (Withdrawal) (*n *= 9). dP/dt max and min (mmHg/sec), maximal acceleration and declaration of pressure in the cardiac cycle; PRSWi (mmHg), slope of preload recruitable stroke work index; Tau (ms), time constant of isovolumetric ventricular relaxation; SW (ml/mmHg), stroke work; EDP (mmHg), end diastolic pressure; Ees (mmHg/ml), end systolic elastance; EDPVR (mmHg/ml), end diastolic pressure volume relationship. All values are obtained by left ventricle pressure-volume catheter at steady state or vena cava occlusions (PRSWi, Ees and EDPVR). *Significant difference from (IR); †significant difference between (AVP-90 mmHg) and (Withdrawal).

The repetitive ischemia/reperfusion (IR) protocol was characterised by a 17% reduction in cardiac output (Figure 1). This was solely a consequence of a pronounced reduction in stroke volume since heart rate increased from 89 ± 7 to 114 ± 6 (p = 0.013) (Figure 2). The fall in systemic blood pressure from 87 ± 3 mmHg at baseline to 67 ± 4 mmHg (p = 0.001) after IR, was meditated by this reduction in CO since vascular resistance and central venous pressures were unaffected (Figures 2 and 3). Furthermore, LAD, carotid and hepatic arterial blood flow dropped from 47 ± 5, 257 ± 23 and 65 ± 12 ml to 42 ± 4, 195 ± 34 and 47 ± 11 ml. This flow reduction was only significant for carotid flow (p = 0.013). Arterial blood flow to the kidney remained constant despite the reduced CO following ischemia (Figure 1). In addition, systemic hypoperfusion was evident from an increased oxygen extraction (art O_2 _sat-SVO_2_) and a reduced base excess (Figure 4). Lactate increased significantly only in the coronary sinus (Table 1). No significant troponin T release occurred. Myocardial contractility was depressed by the ischemia/reperfusion protocol shown by a drop in dP/dt max from 1 566 ± 85 mmHg/sec to 985 ± 74 mmHg/sec (p < 0.001) (Figure 5). Neither end systolic elastance (Ees), nor pre-load recruitable stroke work index (PRSWi) were significantly altered by ischemia (p = 0.31, p = 0.16). The reduced left ventricular function was associated with a trend towards increase in mean pulmonary artery pressure (20 ± 1 mmHg to 23 ± 1 after ischemia (p = 0.074) (Figure 2)). Finally, the diastolic function was modestly affected by the ischemia/reperfusion protocol, as seen by a reduction in dP/dt min from -1 704 ± 82 mmHg/sec to -1 172 ± 78 mmHg/sec (p < 0.001). However, Tau and ventricular compliance (EDP and EDPVR) were unaffected (Figure 5).

**Table 1:** Plasma levels of various metabolites and hormones

	**Baseline**	**IR**	**AVP-initial**	**AVP-90 mmHg**	**Withdrawal**
Lactate (heart) (mM)	0.63 ± 0.12*	1.30 ± 0.26	1.12 ± 0.22	1.44 ± 0.59	1.17 ± 0.21
Lactate (mM)	0.73 ± 0.09	1.26 ± 0.30	1.36 ± 0.18	1.79 ± 0.25	1.21 ± 0.14
Troponin T (μg/L)	0.04 ± 0.02	0.22 ± 0.17	0.27 ± 0.17	0.41 ± 0.24	0.57 ± 0.40
AVP (pg/mL)	82 ± 17	63 ± 21	490 ± 63*	744 ± 178*	116 ± 31†
Epinephrine (nM)	0.33 ± 0.15	0.86 ± 0.44	0.27 ± 0.09	0.13 ± 0.06*	0.24 ± 0.10
Norepinephrine (nM)	0.81 ± 0.17	1.40 ± 0.45	0.50 ± 0.15*	0.48 ± 0.09*	0.92 ± 0.31

Plasma levels of lactate, troponin T, AVP and catecholamines were obtained at different time points in the experiment. See text for experimental details (*n *= 7–9). IR, ischemia-reperfusion; *significant difference from IR; †significant difference between (AVP-90 mmHg) and (Withdrawal).

### Effect of AVP treatment

All haemodynamic responses were evident within the first minutes of AVP infusion and levelled of to the new steady state within the first 20 min. These indices remained stable during the 20 min prior to the recording. The only delayed vasoconstrictive effect was observed in the carotid artery (delayed for approximately 5 min; unpublished results).

Intravenous infusion of AVP caused a further drop in cardiac output from 2.3 ± 0.2 L to 1.7 ± 0.2 L (p < 0.001). This was an effect of the induced bradycardia since HR simultaneously dropped from 114 ± 6 beats/min to 88 ± 6 beats/min (p = 0.004) whereas stroke volume remained unaffected (22 ± 2 ml IR versus 19 ± 2 ml AVP-initial, not significant). AVP had a pronounced effect on systemic vascular resistance, which increased twofold during drug administration. In fact, the vasoconstriction caused by AVP resulted in a normalisation of systemic pressure from 67 ± 4 mmHg to 93 ± 4 mmHg despite the reduction in CO (Figure 2). AVP administration caused vasoconstriction in several vascular beds, that is, a reduction of flow in LAD, carotid, and renal arteries of 23, 18 and 34%, respectively (p = 0.008, p = 0.033, p = 0.001) (Figure 1). This was not observed in the hepatic artery as the blood flow increased from 47 ± 11 ml to 112 ± 19 ml by AVP infusion (p = 0.002). The reduced CO and peripheral vasoconstriction escalated the tissue hypoperfusion shown by a further reduction in SVO_2 _and sin cor O_2 _sat from 49 ± 4 and 29 ± 1% to 42 ± 4 and 21 ± 3% (p = 0.05, p = 0.012), respectively (Figure 4). The lowest measured sin cor O_2 _sat measured was 10% during AVP infusion. Plasma AVP increased after exogenous administration of the hormone confirming an adequate dosage to reach pharmacological levels (Table 1). Concomitantly, the circulation levels of catecholamines fell in the same timeframe. AVP had no effect on contractile function as seen by an unaffected dP/dt max and PRSWi. The Ees value increased from 2.8 ± 0.5 mmHg/ml to 4.3 ± 0.8 mmHg/ml (p = 0.007). Furthermore, there was an impairment of diastolic function induced by AVP infusion. Isovolumetric relaxation time (Tau) increased by 48% (p < 0.001), whereas ventricular compliance was reduced as evident by an increased EDP and EDPVR (Figure 5). An individual adjustment of AVP infusion rate guided by a MAP of 90 mmHg did not principally alter any of the parameters.

### Withdrawal of AVP

By 40 min after AVP infusion was stopped, most of the indices had returned to pre-AVP levels (Tau, EDP, EDPVR, HR, CO, carotid flow, LAD flow, renal flow, hepatic flow, MAP, SVO_2_, sin cor O_2 _sat and SVR). However, base excess remained at values comparable to AVP intervention.

## Discussion

The most striking observation in the present study was an immediate drop in coronary and renal blood flow after administering AVP. Only minutes following the onset of AVP infusion, normal blood pressure was re-established due to a twofold increase in SVR. This was consistent with a twofold increase in organ specific vascular resistance in the brain, heart and kidney. The increased coronary resistance induced by AVP was similar to previous observations in vivo [7,22] and ex vivo [23,24]. Interestingly, AVP caused a reduction in coronary blood flow, despite an increased perfusion pressure, to such extent that it seems to override metabolic flow regulation in the heart. This was supported by a drop in oxygen saturation in blood drawn from the great cardiac vein. This potentially deleterious effect of AVP on myocardial oxygen delivery is in line with a previous case report [25] and studies of felypressin infusions in dogs [7]. A partly compensatory response to the vasoconstriction could be observed in the pigs, as the heart rate and systemic catecholamines fell after AVP infusions.

AVP at this therapeutic level further impaired post-ischemic blood flow to the brain and kidney by vasoconstriction of the respective vascular beds, which is a matter of some concern. This is somewhat in contrast to observations of similar AVP doses in another pig model including septic shock [26]. Malay and co-workers [26] reported a heterogenic vasoconstriction with increased CO and carotid flow and dilated coronary arteries whereas renal flow dropped compatible with our observations. The explanation for the divergent findings of AVP effects on various systemic vascular beds is not known, and whether the vasoactive response to AVP is fundamentally different in sepsis and cardiac ischemia is also not clear. In our study, LAD flow, renal flow and cardiac output fell abruptly, whereas carotid flow had a transient increase during the course of several minutes before the flow dropped below pre-AVP values. This trend (a non sustained cerebral blood flow increase with AVP) was also seen in a previous study during cardiopulmonary resuscitation (CPR) in pigs [27]. The authors did not, however, measure the pre-CPR blood flow in their study, making comparisons difficult. Blood flow in the hepatic artery increased during AVP treatment consistent with a reduction in vascular resistance. However, portal vein blood flow was not measured, and the total hepatic blood flow could therefore have been reduced by the AVP infusion [28,26,29].

There has been some debate regarding the effect of AVP on cardiac contractility. Studies in isolated hearts [30], papillary muscle [31] and myocytes [32,33] have reported a possibly positive inotropic effect. By contrast, studies in intact animals have shown a general trend towards negative effects on contractility [34-36]. However, a major criticism against earlier in vivo studies has been that the use of unphysiologically high doses of AVP may cause myocardial ischemia by coronary vasoconstriction [36]. In the present study, with therapeutically AVP levels, AVP had no effect on cardiac contractility, evident from unchanged dP/dt max and PRSWi. Interestingly however, there was a diastolic dysfunction following AVP infusions, evident from reduced isovolumetric relaxation (Tau) and ventricular compliance (EDPVR and EDP). Also, the increased EES indicate an excessive load dependence in the left ventricle and a manifestation of early contractile failure [36]. These effects are probably due to the increased afterload induced by AVP.

We infused AVP in the lowest dose a giving a significant response in blood pressure. This approach led to plasma levels similar to what have been observed in experimental haemorrhagic or endotoxemic shock [14]. Also, these levels have been observed in patients on extracorporeal circulation [37] with no infusion of AVP. Unfortunately, in the clinical studies on patients in cardiogenic shock indicating a beneficial effect of AVP [6,5], no AVP levels after drug infusion were reported. The drug levels used in these studies (3.6 U/h and 4 U/h) were approximately five times lower compared to AVP levels used in our experimental study. The discrepancy between the experimental and clinical studies indicates that much is still unknown about AVP levels and drug effects in different types of cardiac related shock states [38].

### Limitations

The dose of AVP used in this study was derived from a dose-response study (0.0001 U/kg/min to 0.1 U/kg/min), and the lowest dose giving a significantly increased MAP was chosen for the actual protocol. Comparing this dose (0.005 U/kg/min) to previous clinical studies, it seems that the pigs in our study have a dose-response profile different from humans. It is therefore evident that the dose used in our study should not be applied in clinical practice.

The ischemia reperfusion protocol in our study creates an ischemic cardiac dysfunction where all haemodynamic parameters remain stable for several hours [17]. We used this model to investigate the effects of AVP under stable conditions. With regards to the clinical application of AVP in cardiogenic shock, it would be of great interest to examine the effects of AVP in more advanced acute heart failure. However, haemodynamic studies in an unstable and deteriorating circulation such as cardiogenic shock are difficult to conduct and the data equally hard to interpret when large animal variations occur. Further studies should be undertaken to address the role of AVP in treatment of advanced circulatory shock though our study concurs with previous studies showing pronounced vasoconstrictive properties of AVP [7-13]. It should be mentioned that in contrast to our experimental study, AVP is never administered as monotherapy in patients with acute heart failure, and the aspects of drug interactions and synergy might alter the AVP response in multidrug treatments.

### Clinical implications

The haemodynamic effects of therapeutic level AVP raise serious concerns about the therapeutic potential in patients with attenuated organ perfusion due to acute ischemic heart failure. The negative spiral in cardiogenic shock could possibly be hastened by applying AVP. Therefore, the drug should as a rule not be applied in such patients [39]. Of particular importance, the drug should be used with extreme caution in cardiac surgical patients due to the pronounced vasoconstrictive effect on vascular grafts [37].

## Conclusion

The main observations in this study were that AVP, at therapeutic levels, reduces cardiac output, carotid-, renal- and myocardial blood flow by increased vascular resistance after experimental acute cardiac ischemia. This contributes to an aggravation of systemic hypoperfusion reflected by a reduced oxygen saturation of venous blood from systemic and heart veins. Furthermore, infusing AVP in these dosages resulted in an impairment of the diastolic left ventricular function, whereas myocardial contractility was unaffected by the drug. Finally, these effects were abolished and the pigs returned to pre-AVP values 40 min after withdrawal of the drug.

## Key messages

Vasopressin in therapeutic level impairs blood flow to the brain, heart and kidney in pigs with post-ischemic cardiac dysfunction. The vasoconstriction induced by vasopressin leads to an increased oxygen extraction in the coronary circulation. This indicates that the constrictive effect overrides the myocardial metabolic regulation of the coronary circulation.

Vasopressin has no effect on cardiac contractility, and vasopressin infusion hampers left ventricular diastolic function, probably related to an elevated afterload.

## Competing interests

The authors declare that they have no competing interests.

## Authors' contributions

SM, OJH and TM played a pivotal role in planning and carrying out the experiments as well as writing the manuscript. SEH and TAS contributed in scientific discussions as well as preparing the manuscript. GS planned and carried out the catecholamine analysis.
